# Associations Between Variations in Accumulated Workload and Physiological Variables in Young Male Soccer Players Over the Course of a Season

**DOI:** 10.3389/fphys.2021.638180

**Published:** 2021-03-18

**Authors:** Hadi Nobari, Ana Ruivo Alves, Filipe Manuel Clemente, Jorge Pérez-Gómez, Cain C. T. Clark, Urs Granacher, Hassane Zouhal

**Affiliations:** ^1^Department of Physical Education and Sports, University of Granada, Granada, Spain; ^2^HEME Research Group, Faculty of Sport Sciences, University of Extremadura, Cáceres, Spain; ^3^Sports Scientist, Sepahan Football Club, Isfahan, Iran; ^4^Department of Arts, Humanities and Sport, Polytechnic Institute of Beja, Beja, Portugal; ^5^Research Center in Sport Sciences, Health Sciences and Human Development, Vila Real, Portugal; ^6^Escola Superior Desporto e Lazer, Instituto Politécnico de Viana do Castelo, Viana do Castelo, Portugal; ^7^Centre for Intelligent Healthcare, Coventry University, Coventry, United-Kingdom; ^8^Division of Training and Movement Sciences, University of Potsdam, Potsdam, Germany; ^9^Movement, Sport, Health and Sciences Laboratory (M2S), University of Rennes 2, Rennes, France

**Keywords:** internal load, heart rate, linear sprint, aerobic power, football

## Abstract

This study sought to analyze the relationship between in-season training workload with changes in aerobic power (VO_2m__*ax*_), maximum and resting heart rate (HR_*max*_ and HR_*rest*_), linear sprint medium (LSM), and short test (LSS), in soccer players younger than 16 years (under-16 soccer players). We additionally aimed to explain changes in fitness levels during the in-season through regression models, considering accumulated load, baseline levels, and peak height velocity (PHV) as predictors. Twenty-three male sub-elite soccer players aged 15.5 ± 0.2 years (PHV: 13.6 ± 0.4 years; body height: 172.7 ± 4.2 cm; body mass: 61.3 ± 5.6 kg; body fat: 13.7% ± 3.9%; VO_2m__*ax*_: 48.4 ± 2.6 mL⋅kg^–1^⋅min^–1^), were tested three times across the season (i.e., early-season (EaS), mid-season (MiS), and end-season (EnS) for VO_2m__*ax*_, HR_*max*_, LSM, and LSS. Aerobic and speed variables gradually improved over the season and had a strong association with PHV. Moreover, the HR_*max*_ demonstrated improvements from EaS to EnS; however, this was more evident in the intermediate period (from EaS to MiS) and had a strong association with VO_2m__*ax*_. Regression analysis showed significant predictions for VO_2m__*ax*_ [*F*_(__2,__20)_ = 8.18, *p* ≤ 0.001] with an *R*^2^ of 0.45. In conclusion, the meaningful variation of youth players’ fitness levels can be observed across the season, and such changes can be partially explained by the load imposed.

## Introduction

Soccer, an intermittent-activity profile sport, integrates explosive activities that require high power output of the lower limb muscles (e.g., sprints, changes of direction, jumps), being interspersed by low-intensity activities with brief recovery intervals (Mohr et al., 2005; Bradley et al., 2014). Young soccer players cover distances of 10–13 km throughout matches and achieve around 1,300 individual activities (every 4–6 s), such as accelerations/decelerations (>2 m/s^2^) and changes of direction, with more than 50 turns per match (Mohr et al., 2005; Dalen et al., 2016). Based on antecedence in the literature, the capacity to perform fast and powerful movements in team sports, specifically in soccer, represents one of the most important skills to develop during training in order to improve performance (Zamparo et al., 2015; Christopher et al., 2016). Indeed, the implementation of neuromuscular training (i.e., strength, plyometric, sprint training) in youth is effective in promoting the athletic development and lowering the risk of injuries, due to the increased plasticity of the neuromuscular system, before, during, and after the period of peak height velocity (PHV) (Rumpf et al., 2013).

In addition to physical performance, soccer technical performance requires the progressive development of neuromuscular abilities in match-related power activities, such as sprinting and jumping (Barnes et al., 2014). However, over periods of high exposure to endurance training (e.g., preseason), improving these abilities presents a challenge, largely because training-induced power and speed adaptations might be impaired by concurrent training (Loturco et al., 2015; Noon et al., 2015). In fact, evidence regarding the interference effect in professional soccer players and elite youth players is well-reported (Loturco et al., 2015; Noon et al., 2015). However, the findings in young players are not well-described. Accordingly, it is necessary to better understand potential interference effects of concurrently performed power and endurance training to optimize players’ physiological adaptations over the soccer season using individualized training load data. Indeed, this knowledge could be helpful to short- and long-term coaching decision-making (Barker and Armstrong, 2010; Álvarez-Kurogi et al., 2019).

As recognized in the literature, there are various key points related to training effectiveness, such as the period of the season (e.g., greater fitness variations are described in pre-season compared to in-season), the players’ competitive level (e.g., amateurs report superior adaptations than elite players succeeding soccer actions), and the baseline level or the training load imposed (Caldwell and Peters, 2009; Kalapotharakos et al., 2011; Coratella et al., 2016; Clemente et al., 2019). Interestingly, studies based on the analysis of performance during the season have predominantly focused on professional and semiprofessional adult players (Haritonidis et al., 2004; Caldwell and Peters, 2009), yet there is a distinct dearth of studies involving young players (Buchheit et al., 2010; Dragijsky et al., 2017). In fact, managing physical fitness parameters in younger ages is fundamental to the training process, because it is an imperative period of physical development (Lloyd and Oliver, 2012). Thus, it is pertinent that researchers and coaches understand the factors that might affect performance to improve the training process. This will provide useful information on young players’ strengths and weaknesses over the season.

In sports practice, the physical fitness level is specific for each individual, and the accurate assessment is imperative to achieve training progress by enabling physiological adaptations (McArdle et al., 2011). Accordingly, heart rate frequency, maximum oxygen uptake (VO_2m__*ax*_), and the individual anaerobic threshold are useful indicators of cardiorespiratory fitness (McArdle et al., 2011). Previous studies have examined the effects of periodized training on physical fitness (i.e., aerobic power, speed, repeated sprint ability) in soccer players (Owen et al., 2012; Fitzpatrick et al., 2018). However, there is a lack of knowledge regarding the physiological responses to a given training load in young soccer players (Fitzpatrick et al., 2018).

In terms of training load imposed, it is proposed that the load should be sufficient to stimulate and increase players’ physical fitness (Impellizzeri et al., 2005). Indeed, given that variegated training loads may yield different adaptations in players, it is important to monitor load, in order to identify possible relationships between the dose and the effect. Although changes in physical fitness variables, preceded by specific training periods, are evident in soccer players, the association between such changes and the accumulated load needs to be further investigated (Clemente et al., 2019). There is only one study available (Clemente et al., 2020a) that has examined the relationship between cardiorespiratory fitness and the accumulated training load parameters over a 4-months in-season period. Results from Clemente et al. (2020a) indicated that training effort is strongly associated with changes in aerobic power. Indeed, no study, to the authors’ knowledge, has examined the relationship between training load, maturity status, and different physiological variables (e.g., VO_2m__*ax*_, maximum and resting heart rate [HR_*max*_ and HR_*rest*_], and speed variables) in elite young male soccer players. Moreover, the relationship between training load during the competitive season and performance is very important to facilitate improvements in strength and conditioning practices. For this reason, the aim of this study was to analyze the relationships between maturity status and training load with variations in physiological variables in soccer players younger than 16 years (under-16 [U16]). Based on the relevant literature (Clemente et al., 2020b; Nobari et al., 2020b), we hypothesized that the accumulated training load might partially explain variation of youth players’ fitness levels across the soccer season.

## Materials and Methods

### Participants

The study sample consisted of 23 young male sub-elite soccer players who compete at a national U16 level. Players’ mean chronological ages were 15.5 ± 0.2 years, VO_2m__*ax*_, 48.5 ± 2.6 mL⋅kg^–1^⋅min^–1^, and all players were post-PHV (+ 1.9 ± 0.3 years) (Table 1). Among the 23 participants, there were nine defenders, six midfielders, four wingers, and four forward. Inclusion criteria were players (i) who participated in at least 90% of training sessions throughout the season, (ii) did not use dietary supplements during the study, (iii) who were uninjured over the course of the study, and (iv) who did not participate in another training program along with this study. Each player who did not participate in a match during the week participated in a separate training session as a replacement. Exclusion criteria comprised (i) goalkeepers, due to the different variations in the physical demands with outfield players. Prior to the start of the study, explanations on the goals and procedures of the study were provided to all participants and their parents/legal representatives. All players were informed about potential risks and benefits of participating in the study. The study was conducted in accordance with the latest version of the Declaration of Helsinki and approved by the Ethics Committee of the University of Isfahan with the ethical code number; IR.UI.REC.1397.181. Prior to study commencement, all players and their parents/guardians signed a written informed consent form.

**TABLE 1:** Descriptive characteristics of 23 soccer players under 16.

**Variables**	**Mean** ± **SD**	**Confidence interval 95%**
Body height (cm)	172.7 ± 4.2	(171–174.4)
Body mass (kg)	61.3 ± 5.6	(59–63.6)
Sitting height (cm)	96.6 ± 2.1	(91.8–93.4)
Age at PHV (years)	13.6 ± 0.4	(13.5–13.7)
Maturity offset (years)	1.9 ± 0.3	(1.7–2.0)
Chronological age (years)	15.5 ± 0.2	(15.3–15.5)
Training experience (years)	6.2 ± 1.6	(5.6–6.9)
VO_2m__*ax*_ (mL kg^–1^⋅min^–1^)	48.4 ± 2.6	(47.3–49.4)
Body fat (%)	13.7 ± 3.9	(12.1–15.3)

*PHV, peak height velocity; VO_2m__*ax*_, maximal oxygen consumption.*

### Experimental Approach to the Problem

**FIGURE 1:**
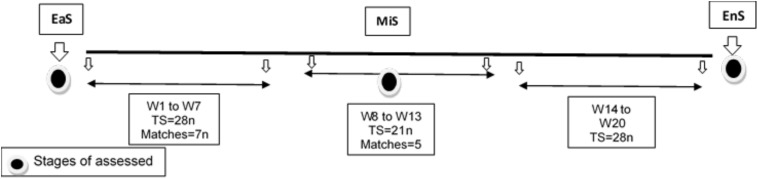
Research outline of the monitoring on training, match, and assessed sessions during the competition season. EaS, early season = before league for assessments and W1–W7 for the cohort study; MiS, midseason = mid league for assessments and W8–W13 for the cohort study; EnS, end-season = after league for assessments and W14–W20 for the cohort study; W, week; TS, training sessions.

The present study was performed during the competitive season, lasting 20 weeks, and included three stages of evaluation (before, mid, and after the end of the season). The season was divided into three periods: (i) early-season (EaS) = week (W) 1 to W7; (ii) mid-season (MiS) = W8–W13; and (iii) end-season (EnS) = W14–W20 according to Figure 1. The first assessment was in the week before the start of the season, the second test point was in the 11th week of the mid-season, and the third assessment was in the week after the season ended. During each test, players were measured on consecutive days. Individual indicators of each player were assessed on the first test day (i.e., anthropometric and body composition variables) and maturity status to determine age at PHV. On the next test, the linear sprint short test (LSS), the linear sprint medium test (LSM), and the intermittent fitness test 30–15 (30–15_*I*__*FT*_) were performed. Evaluations of each stage were performed at the same time, temperature, and humidity (Medicine ACoS, 2013). Maximal heart rate (HR_*max*_) was also recorded during the 30–15_*I*__*F*__*T*_ test. Resting heart rate (HR_*rest*_) was measured in lying position 1 day after resting when waking up in the morning. A familiarization session was scheduled 1 week prior to the assessment. For this cohort study, 30 min after each training session, all players reported load of training, and then each “training load” was calculated, alongside training time, to discern accumulated workload for any period. Each week, the training days included the following: (i) technical and skills with moderate-intensity aerobic training; (ii) tactical and full body-weight training; (iii) high-intensity training or small side games; (iv) tactical with agility and coordination training; (v) and finally, an official or friendly match. Athletes were kindly asked not to change their diet throughout the study period.

### Anthropometrics and Body Composition

Stature was measured with a stadiometer [Seca model 213, Germany (accuracy of ± 5 mm)]. Participants stood barefoot and touched their lower back, head, shoulder blades, buttocks, and heel to the stadiometer, with their feet placed together. After adopting the correct position, height was measured vertically by the rod from above the head. To measure the sitting height, participants were required to sit on a 50-cm-height table (forward face) with their lower back as close as possible to the stadiometer. They kept their upper body straight and put their hands on their feet. Then, the height measured between the highest point of the head (vertex) and the (ischial spines) plane where they were sitting. Body mass was measured using weighing scales [Seca model 813, United Kingdom (accuracy ± 0.1 per kg)]. Each subject, with light clothes, stood, and the scale recorded the subjects’ mass. The technical error of anthropometric measurements, interobserver and intraobserver, with the same observer was less than 3% (Nobari et al., 2020a,b). A practical method for the determination of maturity offset was applied using three somatic dimensions (height, sitting height, leg length) and chronological age as well as their interactions: [maturity offset = −9.236 + 0.0002708 (leg length × sitting height) −0.001663 (chronological age × leg length) + 0.007216 (chronological age × sitting height) + 0.02292 (mass by height ratio)]. This equation has previously been validated by Mirwald et al. (2002).

The subcutaneous fat thickness of the triceps and subscapular points of the body were measured. Percentage body fat was estimated using the equation of Slaughter et al. (1988). Skin thickness was obtained by using calibrated Lafayette skinfold calipers (United States, with an accuracy of ± 0.1 mm). All measurements were performed twice on the right side of the body; the final score was recorded as the mean of two measurements. If technical error was higher than 5%, the measurements were executed again, and the median value was recorded (Nobari et al., 2020a,b). All anthropometric and body composition measurements were taken by a skilled practitioner, with a 5-year history in this area, and all measurements were assessed in the morning (Arazi et al., 2015; Rahmat et al., 2016).

### Monitoring Internal Training Loads

Players were monitored daily for their rating perceived of exertion (RPE) using the Category-Ratio-10 Borg’s scale, which is a valid and reliable scale to estimate the intensity of a session (Foster et al., 2001). To the question, “How intense was your session?” players answered in the interval range of 1 (minimum effort) and 10 (maximal effort). Players answered 30 min after the end of each training session. Additionally, the duration of the training sessions, in minutes, was recorded. As a measure of internal load, the session RPE was calculated by multiplying the score in Category-Ratio-10 scale (11 numbers) by the duration of the session in minutes. Players were previously familiarized with the scale, having used it in the previous 2 years in the club. In this study, the accumulated load (for training and competition) was used for 20 weeks. These weeks of the full-competition season were divided into three periods: early-season (EaS), W1–W7; mid-season (MiS), W8–W13; and end-season (EnS), W14–W20, for analysis used the mean of the accumulated load of periods (MALP).

### Intermittent Fitness Test 30–15 (30–15_*I*__*FT*_)

The 30–15_*I*__*FT*_ was used to calculate the VO_2m__*ax*_ of the players. The test started at an average speed of 8 km⋅h^–1^ between a 40 m shuttle. Players started running 30 s after hearing the first beep (from line A to B and from B to C and then back). Each stage consisted of running for 30 s and then resting for 15 s. Thereafter, every stage had a 0.5 km⋅h^–1^ increment until volitional exhaustion (Buchheit et al., 2010). Each person’s score was the last stage of speed that he could not continue, or three times not making it to the 2 m lines. At this time, the final velocity of the IFT (VIFT) was recorded. All these steps were performed after a standardized warm-up of 10 min. The following equation was used to determine the VO_2m__*ax*_ (mL⋅kg^–1^⋅min^–1^) of each player (Buchheit et al., 2010) = 28.3 −(2.15 × 1) −(0.741 × age) −(0.0357 × mass) + (0.0586 × age × VIFT) + (1.03 × VIFT). The test–retest reliability was calculated, using intraclass coefficient (ICC), to be 0.86.

### Heart Rate Measurements

The HR variables were measured using Mi-Band 3, Xiaomi Company, made in China. The HR_*max*_ was recorded during the IFT_30__–__15_ test, to measure each player’s HR_*rest*_ at each assessment stage. In the morning, when waking up, they performed three assessments over 3 days. Information was received by the researcher every day, and the average of 3 days was considered as a criterion for analysis.

### Linear Sprint Test

To measure linear sprint performance, the LSS 10 m and the LSM 20 m tests were used. These two variables were performed with the Newtest Powertimer 300-series device made in Finland, and times were recorded. Each distance was repeated three times with 3 min of rest. The best time in each distance was considered as a criterion for analysis. The ICCs in these tests was LSS = 0.91 and LSM = 0.94. The photocells were adjusted from the athlete’s pelvis height. All players followed a standardized warm-up of 10–15 min, including jogging, running drills (e.g., high knee, butt kicks, carioca), and two to three linear sprints. After warming up, the players stood at a distance of 70 cm from the start line, and then, on the command of the instructor, the player started covering the specified distance at full speed (Mirkov et al., 2008).

### Statistical Analyses

Based on a previous work that highlighted large to very large correlations between physiological variables in soccer players (Clemente et al., 2019), we computed the sample size necessary to achieve a power of, at least, 0.90. Accordingly, using a two-tailed hypothesis, expected large to very large correlations, and an α error probability of 0.05, 21 participants would be required to achieve the aforementioned power. The normality of the data was checked by Shapiro–Wilk test. Data are presented as means and standard deviations. In cases where data were normally distributed, Pearson correlation coefficients were calculated for measures of physical fitness and training load used for normal data (i.e., HR_*max*_ and MALP of between periods), and if normality of data was violated, Spearman correlation coefficients were computed. The following threshold was used to determine the amplitude of correlation levels (Batterham and Hopkins, 2006): < 0.1 = trivial, 0.1–0.3 = small, 0.3–0.5 = moderate, 0.5–0.7 = large, 0.7–0.9 = very large, and > 0.9 = nearly perfect. Multiple linear (least squares) regression analysis was calculated to predict the percentage of change in fitness levels (i.e., VO_2m__*ax*_, HR, and speed variables) based on accumulated load, baseline levels, and PHV. In accordance with previous recommendations (Jenkins and Quintana-Ascencio, 2020), the Akaike information criterion (AIC) for each model’s regression was additionally calculated, to support inferences about the model’s suitability. We analyzed the ICCs for the reliability evaluated for within-session outcome of all measures but used the 30–15_*I*__*F*__*T*_ test–retest reliability. The ICC > 0.7 was suitable (Baumgartner and Chung, 2001). Statistical significance was accepted, *a priori*, at *p* < 0.05. All statistical analyses were performed using GraphPad Prism 8.0.1 software, except for multiple linear regression and AIC, which were calculated using R software (version 1.2.5019).

## Results

**FIGURE 2:**
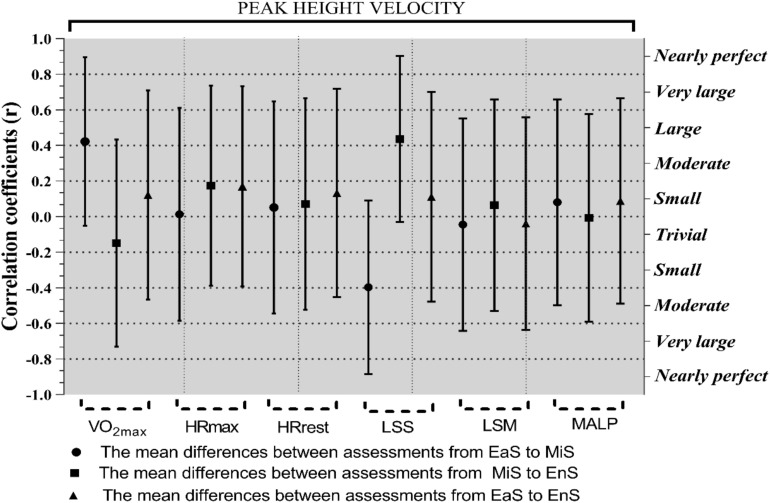
Correlation coefficients (95% CI) the peak height velocity soccer player with the VO_2m__*ax*_ (mL⋅kg^–1^⋅min^–1^), maximal oxygen consumption; HR_*max*_ (bpm), maximal heart rate; HR_*rest*_ (bpm), resting heart rate; LSS (s), linear sprint short test; LSM (s), linear sprint medium test; MALP (A.U.), the mean of the accumulated load of period; A.U., arbitrary unit.

Figure 2 shows the correlation coefficient between PHV with mean differences between assessments and the accumulated load of between periods at the 95% confidence interval (EaS to MiS, MiS to EnS, and EaS to EnS). There were significant correlations between VO_2m__*ax*_ at EaS to MiS (*r* = 0.49; 95% CI, 0.09–0.76; *p* = 0.02), LSS at EaS to MiS (*r* = −0.46; 95% CI, −0.74 to −0.05; *p* = 0.02), and LSS at MiS to EnS (*r* = 0.51; 95% CI, 0.11–0.76; *p* ≤ 0.001) with PHV. Likewise, VO_2m__*ax*_ at EaS to EnS (*r* = 0.67 large; 95% CI, 0.35–0.85; *p* ≤ 0.001) and HR_*max*_ at EaS to MiS (*r* = 0.46; 95% CI, 0.052–0.74; *p* = 0.03) were associated with VO_2m__*ax*_ at MiS to EnS, respectively. In addition, HR_*max*_ at EaS to MiS (*r* = 0.48; 95% CI, 0.07–0.75; *p* = 0.02) was related to VO_2 max_ at EaS to EnS. For HR_*max*_, there were strong associations between EaS to EnS and MiS to EnS (*r* = 0.94; 95% CI, 0.86–0.95; *p* ≤ 0.001). Further, HR_*rest*_ at EaS to EnS was related to HR_*rest*_ at EaS to MiS (*r* = 0.41; 95% CI, −0.01 to 0.72; *p* = 0.05), and MiS to EnS (*r* = 0.75; 95% CI, 0.48–0.89; *p* ≤ 0.0001), respectively.

Overall, the relations MALP at EaS to MiS with HR_*max*_ at EaS to EnS (*r* = −0.42; 95% CI, 0.01–0.71]; *p* = 0.047) and MALP at MiS to EnS (*r* = −0.69; 95% CI, −0.86 to −0.38]; *p* ≤ 0.001) were moderate and large (Table 2).

**TABLE 2:** Pearson and Spearman correlation analysis between the mean differences between assessments and the average accumulated load of between periods.

**Variable**	**β0**	**β1**	**β2**	**β3**	**β4**	**β5**	**β6**	**β7**	**β8**	**β9**	**β10**	**β11**	**β12**	**β13**	**β14**	**β15**	**β16**	**β17**	**β18**	**β19**	**β20**	**β21**
PHV (β0)	1.00																					
VO_2m__*ax*_ 1 (β1)	**0.49**	1.00																				
VO_2m__*ax*_ 2 (β2)	−0.18	−0.36	1.00																			
VO_2m__*ax*_ 3 (β3)	0.15	0.31	**0.67**	1.00																		
HR_*max*_ 1 (β4)	0.02	0.15	**0.46**	**0.48**	1.00																	
HR_*max*_ 2 (β5)	0.21	−0.05	−0.07	−0.09	−0.38	1.00																
HR_*max*_ 3 (β6)	0.20	0.01	0.01	0.01	−0.04	**0.94**	1.00															
HR_*rest*_ 1 (β7)	0.06	0.05	−0.08	−0.10	0.17	0.10	0.14	1.00														
HR_*rest*_ 2 (β8)	0.09	−0.12	0.05	0.02	0.16	−0.06	0.00	−0.25	1.00													
HR_*rest*_ 3 (β9)	0.16	0.01	−0.03	0.02	0.26	0.07	0.16	**0.41**	**0.75**	1.00												
LSS 1 (β10)	**−0.46**	**−0.57**	0.07	−0.23	−0.27	0.19	0.13	−0.19	−0.23	−0.39	1.00											
LSS 2 (β11)	**0.51**	0.38	0.01	0.21	−0.13	0.17	0.15	−0.25	0.26	0.08	−0.17	1.00										
LSS 3 (β12)	0.13	−0.01	−0.03	−0.03	−0.29	0.22	0.17	−0.33	0.14	−0.10	**0.54**	**0.69**	1.00									
LSM 1 (β13)	−0.05	−0.01	0.33	0.23	0.25	−0.13	−0.10	−0.17	0.00	−0.14	−0.15	0.12	−0.07	1.00								
LSM 2 (β14)	0.08	0.15	−0.16	−0.23	−0.20	−0.01	−0.06	0.29	−0.20	0.06	−0.25	0.22	−0.01	−0.17	1.00							
LSM 3 (β15)	−0.05	0.11	−0.01	−0.09	−0.12	−0.06	−0.11	0.15	−0.25	−0.08	−0.20	0.23	−0.02	**0.44**	**0.72**	1.00						
MALP 1 (β16)	0.10	0.03	0.35	0.31	0.11	0.35	**0.42**	−0.08	0.14	0.11	−0.03	0.24	0.08	0.33	0.20	0.36	1.00					
MALP 2 (β17)	−0.01	0.13	−0.16	−0.04	−0.07	−0.31	−0.36	−0.13	−0.19	−0.24	0.09	−0.15	0.01	−0.21	−0.24	−0.32	**−0.69**	1.00				
MALP 3 (β18)	0.11	0.20	0.22	0.32	0.03	0.01	0.02	−0.27	−0.09	−0.18	0.09	0.09	0.11	0.11	−0.07	0.01	0.30	**0.48**	1.00			
VIFT 1 (β19)	**−0.42**	−1.00	0.10	**−0.59**	−0.19	0.06	−0.01	−0.04	−0.11	−0.13	0.34	−0.25	0.19	−0.02	0.04	0.02	−0.03	−0.13	−0.21	1.00		
VIFT 2 (β20)	0.20	0.10	−1.00	**−0.75**	−0.22	0.07	−0.01	0.09	−0.09	−0.06	0.04	−0.02	0.02	−0.18	0.01	−0.09	−0.35	0.16	−0.22	−0.09	1.00	
VIFT 3 (β21)	−0.12	**−0.59**	**−0.74**	**−1.00**	−0.30	0.10	−0.01	0.05	−0.15	−0.13	0.26	−0.18	0.15	−0.15	0.04	−0.06	−0.30	0.04	−0.32	**0.60**	**0.74**	1.00

*Significant differences (p ≤ 0.05) are highlighted in bold font.*

*PHV, peak height velocity; VO_2m__*ax*_, maximal oxygen consumption; HR_*max*_, maximal heart rate; HR_*rest*_, resting heart rate; LSS, linear sprint short test; LSM, linear sprint medium test; MALP, the mean of the accumulated load of period; VIFT, final velocity of the 30–15_*I*__*FT*_; 1, 2, and 3 = the mean differences between assessments (EaS to MiS, and MiS to EnS and EaS to EnS), respectively.*

**FIGURE 3:**
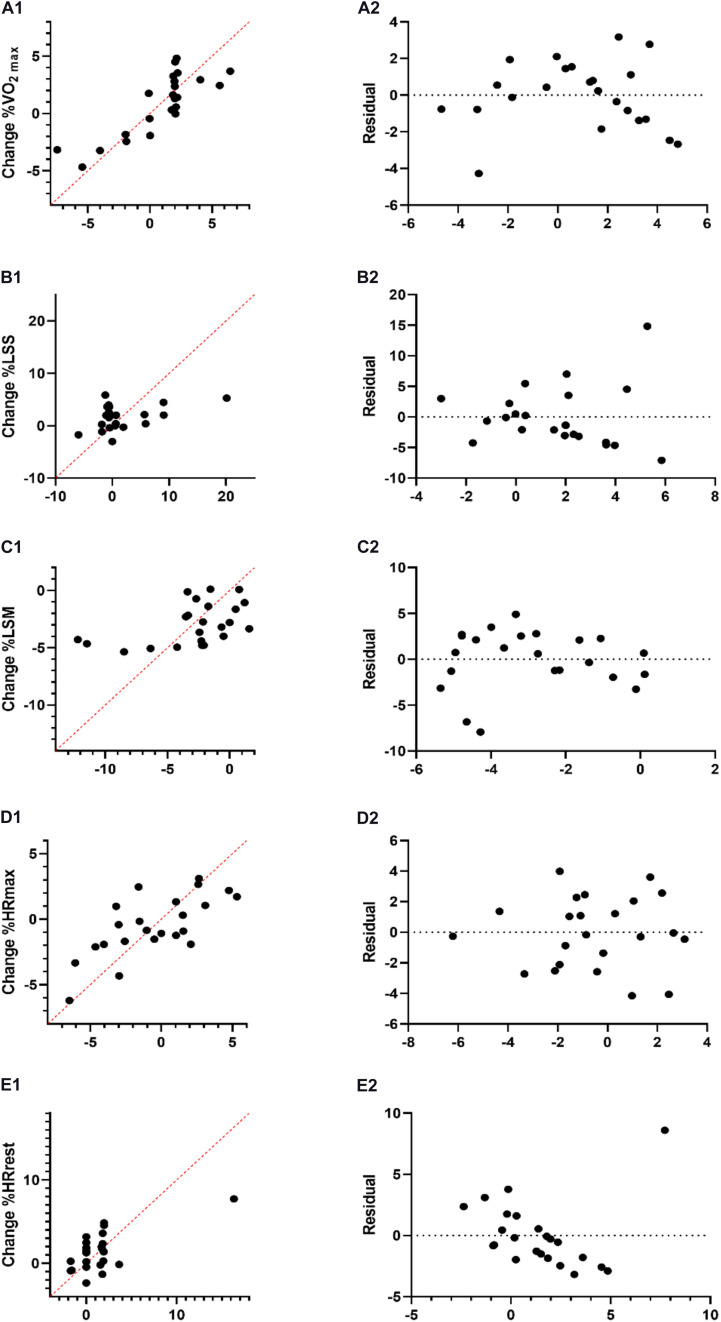
Multiple linear regression analysis was calculated to predict the percentage of change in fitness levels includes: VO_2m__*ax*_, **(A1)**; LSS **(B1)**; LSM **(C1)**; HR_*max*_ **(D1)**; and HR_*rest*_ **(E1)** based on accumulated load, baseline levels, and PHV with residual plot for each analysis VO_2m__*ax*_, **(A2)**; LSS **(B2)**; LSM **(C2)**; HR_*max*_ **(D2)**; and HR_*rest*_ **(E2)**, the difference between the actual value of the dependent variable and the value predicted by the residual provided. VO_2m__*ax*_, maximal oxygen consumption.

Multiple linear regression analyses were performed to predict the percentage of change in fitness levels (i.e., VO_2m__*ax*_, HR, and speed variables) based on accumulated load, baseline levels, and PHV. The regression for VO_2m__*ax*_ was significant [*F*_(__8,__14)_ = 3.72, *p* = 0.02], with an *R*^2^ of 0.68. Participants predicted VO_2m__*ax*_ (*Y*) was equal to β0 + β1 (accumulated load) + β2 (PHV) + β3 (Maturity offset) + β4 (EaS VO_2 max_) + β5 (baseline HRmax) + β6 (EaS HRrest) + β7 (EaS LSS) + β8 (EaS LSM). Where accumulated load was coded or measured as arbitrary units, maturity status coded or measured as years, fitness status coded or measured as mL⋅kg^–1^⋅min^–1^, bpm, and s. Participants predicted VO_2m__*ax*_ levels during the season decreased −0.84 mL⋅kg^–1^ ⋅ min^–1^ for each mL⋅kg^–1^⋅min^–1^ of VO_2m__*ax*_, as well as decreasing −0.30 mL⋅kg^–1^⋅min^–1^ for each bpm of HR_*rest*_, both baseline VO_2m__*ax*_ and HR_*rest*_ were significant predictors of aerobic power levels (Table 3). An additional predictive equation was made for these two variables (VO_2m__*ax*_ and HR_*rest*_), which was significant [*F*_(2, 20)_ = 8.18, *p* ≤ 0.001], with an *R*^2^ of 0.45. Participants’ predicted VO_2m__*ax*_ (Y) was equal to β0 + β1 (VO_2m__*ax*_) + β2 (HR_*rest*_). Participants’ VO_2m__*ax*_ levels during the season decreased −0.89 mL⋅kg^–1^⋅min^–1^ for each mL⋅kg^–1^⋅min^–1^ of VO_2m__*ax*_, as well as decreases −0.17 mL⋅kg^–1^⋅min^–1^ for each bpm of HR_*rest*_ (Table 3). Residual plots are depicted in Figure 3.

**TABLE 3:** Multiple linear regression analysis: percentage of change in VO_2m__*ax*_ with accumulated load, baseline fitness levels, and PHV.

**Variable**	**β**	**Estimate**	**| *t*|**	***P*-value**	**95% CI for estimated**	**Total predict**
VO_2 max_ (%)	β0	37.13	0.67	0.51	−81.9 to 156.1	***R*^2^ = 0.68 Adjusted *R*^2^ = 0.50 *P* = 0.02 AIC = 112.1**
ACL (A.U.)	β1	0.00	0.91	0.38	−0.002 to 0.001	
Maturity offset (years)	β2	2.91	1.23	0.24	−2.17 to 7.98	
PHV (years)	β3	3.96	1.30	0.21	−2.56 to 10.48	
VO_2m__*ax*_ (mL ⋅ kg^–1^ ⋅ min^–1^)	β4	−0.84	3.78	**≤0001**	−1.32 to −0.36	
HR_*max*_ (bpm)	β5	−0.08	0.64	0.53	−0.36 to 0.2	
HR_*rest*_ (bpm)	β6	−0.30	2.08	**0.06**	−0.61 to 0.01	
LSS (s)	β7	5.60	1.74	0.10	−1.31 to 12.50	
LSM (s)	β8	−0.89	0.45	0.66	−5.15 to 3.36	

*Significant differences (p ≤ 0.05) are highlighted in bold font.*

*PHV, peak height velocity; VO_2m__*ax*_, maximal oxygen consumption; HR_*max*_, maximal heart rate; HR_*rest*_, resting heart rate; LSS, linear sprint short test; LSM, linear sprint medium test; ACL, all of accumulated load of 20 weeks;%, percentage of change in between assessments from EaS to EnS; CI, confidence interval; AIK, Akaike information criterion; A.U., arbitrary unit.*

For LSS [*F*_(8, 14)_ = 0.38, *p* = 0.92, *R*^2^ = 0.18] and LSM [*F*_(8, 14)_ = 0.55, *p* = 0.80, *R*^2^ = 0.24], multiple linear regression did not yield any significant findings (Table 4).

**TABLE 4:** Multiple linear regression analysis: percentage of change in speed variables with accumulated load, baseline fitness levels, and PHV.

**Variable**	**β**	**Estimate**	**| *t*|**	***P*-value**	**95% CI for estimated**	**Total predict**
LSS (%)	β0	−94.42	0.65	0.529	−408.2 to 219.3	***R*^2^ = 0.18 Adjusted *R*^2^ =**−**0.29*P* = 0.92 AIC = 156.71**
ACL (A.U.)	β1	0.001	0.48	0.639	−0.003 to 0.004	
Maturity offset (yrs)	β2	5.20	0.84	0.414	−8.18 to 18.58	
PHV (yrs)	β3	5.58	0.7	0.498	−11.60 to 22.76	
VO_2m__*ax*_ (mL kg^–1^ min^–1^)	β4	−0.89	1.51	0.153	−2.15 to 0.37	
HR_*max*_ (bpm)	β5	0.31	0.93	0.371	−0.41 to 1.03	
HR_*rest*_ (bpm)	β6	−0.22	0.59	0.566	−1.04 to 0.59	
LSS (s)	β7	0.11	0.01	0.989	−18.11 to 18.33	
LSM (s)	β8	−3.15	0.60	0.557	−14.37 to 8.08	

**Variable**	**β**	**Estimate**	**| *t*|**	***P*-value**	**95% CI for estimated**	**Total predict**

LSM (%)	β0	−104.2	1.08	0.298	−310.8 to 102.4	***R*^2^ = 0.24 Adjusted *R*^2^ =**−**0.20*P* = 0.80 AIC = 137.48**
ACL (A.U.)	β1	=0001	0.95	0.359	−0.001 to 0.003	
Maturity offset (yrs)	β2	2.56	0.62	0.544	−6.25 to 11.37	
PHV (yrs)	β3	7.65	1.45	0.169	−3.67 to 18.96	
VO_2m__*ax*_ (mL kg^–1^ min^–1^)	β4	0.28	0.73	0.478	−0.5484 to 1.11	
HR_*max*_ (bpm)	β5	0.09	0.41	0.686	−0.38 to 0.57	
HR_*rest*_ (bpm)	β6	0.05	0.21	0.838	−0.49 to 0.59	
LSS (s)	β7	2.06	0.37	0.718	−9.93 to 14.06	
LSM (s)	β8	−3.61	1.05	0.313	−10.99 to 3.78	

*Significant differences (p = 0.05) are highlighted in bold.*

*PHV, Peak height velocity; VO_2m__*ax*_, Maximal oxygen consumption; HR_*max*_, Maximal heart rate; HR_*rest*_, resting heart rate; LSS, Linear sprint short test; LSM, linear sprint medium test; ACL, The accumulated load of 20 weeks; %, The percentage of change in between assessments from EaS to EnS; CI, Confidence interval; AIK, Akaike information criterion; A.U., Arbitrary unit.*

For HR_*max*_ [*F*_(8, 14)_ = 1.75, *p* = 0.17, *R*^2^ = 0.50] and HR_*rest*_ [*F*_(8, 14)_ = 1.30, *p* = 0.32, *R*^2^ = 0.43], multiple linear regression did not yield any significant findings (Table 5).

**TABLE 5:** Multiple linear regression analysis: percentage of change in HR measurements with accumulated load, baseline fitness levels, and PHV.

**Variable**	**β**	**Estimate**	**| *t*|**	***P*-value**	**95% CI for estimated**	**Total predict**
HR_*max*_ (%)	β0	110.3	1.57	0.138	−40.24 to 260.8	***R*^2^ = 0.50**
ACL (A.U.)	β1	≤−0.001	0.85	0.409	−0.002 to ≤ 0.001	**Adjusted**
Maturity offset (years)	β2	−1.46	0.49	0.634	−7.88 to 4.96	***R*^2^ = 0.21**
PHV (years)	β3	−5.44	1.42	0.179	−13.68 to 2.81	***P* = 0.17**
VO_2m__*ax*_ (mL⋅kg^–1^ ⋅ min^–1^)	β4	0.27	0.95	0.357	−0.34 to 0.88	**AIC = 122.93**
HR_*max*_ (bpm)	β5	−0.47	2.93	**0.011**	−0.82 to −0.13	
HR_*rest*_ (bpm)	β6	0.36	1.97	0.069	−0.03 to 0.75	
LSS (s)	β7	−3.12	0.77	0.456	−11.86 to 5.62	
LSM (s)	β8	0.79	0.31	0.759	−4.6 to 6.17	

**Variable**	**β**	**Estimate**	**| *t*|**	***P* value**	**95% CI for estimated**	**Total predict**

HR_*rest*_ (%)	β0	60.38	0.74	0.468	−113.1 to 233.9	***R*^2^ = 0.43 Adjusted *R*^2^ = 0.01 *P* = 0.32 AIC = 129.46**
ACL (A.U.)	β1	≤ 0.001	0.93	0.368	≤−0.001 to 0.003	
Maturity offset (years)	β2	−4.42	1.28	0.221	−11.82 to 3	
PHV (years)	β3	−6.33	1.43	0.175	−15.8 to 3.18	
VO_2m__*ax*_ (mL⋅kg^–1^ min^–1^)	β4	−0.1	0.31	0.765	−0.8 to 0.6	
HR_*max*_ (bpm)	β5	−0.02	0.13	0.901	−0.42 to 0.38	
HR_*rest*_ (bpm)	β6	−0.13	0.62	0.546	−0.58 to 0.32	
LSS (s)	β7	−5.1	1.1	0.296	−15.17 to 4.98	
LSM (s)	β8	3.406	1.18	0.259	−2.79 to 9.61	

*Significant differences (p ≤ 0.05) are highlighted in bold font. PHV, peak height velocity; VO_2m__*ax*_, maximal oxygen consumption; HR_*max*_, maximal heart rate; HR_*rest*_, resting heart rate; LSS, linear sprint short test; LSM, linear sprint medium test; ACL, accumulated load of 20 weeks;%, percentage of change in between assessments from EaS to EnS; CI, confidence interval; AIK, Akaike information criterion; A.U., arbitrary unit.*

*Post hoc* power analysis was conducted to detect the achieved power for each multiple linear regression analysis, based on a two-tailed hypothesis, a sample size of 23, nine predictors, and the achieved effect size. For percentage of change in VO_2m__*ax*_, with accumulated load, baseline fitness levels, and PHV, the achieved power was 0.95. For percentage of change in speed variables (LSS and LSM), with accumulated load, baseline fitness levels, and PHV, the achieved power was 0.68, due to the trivial effect sizes. Finally, for percentage of change in HR measurements (HR_*max*_ and HR_*rest*_), with accumulated load, baseline fitness levels, and PHV, the achieved power was 0.88.

## Discussion

This study sought to discern the relationship between in-season training workload with changes in VO_2m__*ax*_, heart rate, and speed variables in male U16 soccer players. Accordingly, large and moderate correlations were reported between aerobic power, HR, speed variables, and MALP between the different periods of the soccer season. We additionally aimed to explain changes in fitness levels during the in-season through regression models considering accumulated load, baseline levels, and PHV as predictors. Significant predictors (i.e., VO_2m__*ax*_ and HR_*rest*_) of aerobic power levels during the competition season were found, but not for the speed variables (i.e., LSS and LSM), whereas for HR, only during the competition season, HR_*max*_ was reported as a significant predictor of percentage of change in HR_*max*_ levels.

The capacity of soccer players to develop and preserve high physical fitness levels over the season is paramount to success (Krustrup et al., 2005; Bloomfield et al., 2007). The current study indicated that aerobic capacity improved from the EaS to the EnS, with more evident increases from the MiS to the EnS. Moreover, VO_2m__*ax*_ between EaS to MiS was significantly related to PHV. Following the present study, Clemente et al. (2020a) also reported significant improvements in maximal aerobic speed over the season in young soccer players, with the greatest improvements at the EnS (4 months after baseline test). However, Dragijsky et al. (2017) showed an improvement in endurance, with the highest levels reported in the EaS, followed by a partial fall in the MiS, as well as at the EnS. Findings from this study can most likely be explained by the possible changes in anthropometric traits, heart and lung mass, and nervous system maturation associated with normal growth and maturation leading to improvement in aerobic capacity (Malina et al., 2015; Clemente et al., 2020a).

A significant improvement in LSS from EaS to MiS was reported, but greater levels were evident from MiS to EnS and were significantly related to PHV. A differential timing of adolescent spurts body dimensions, functional capacities, and sports skills during youth has been reported (Malina et al., 2015). Indeed, for example, data pertaining to speed suggest peak gains occur before PHV in boys (Beunen et al., 1988), whereas tests of strength and power achieved peak gains after PHV (Beunen and Malina, 1988; Malina et al., 2004), and peak velocity in VO_2m__*ax*_ was reached concomitant with PHV in boys and girls (Mirwald and Bailey, 1987; Geithern et al., 2004). Thus, considering the strong relationship between PHV and speed variables (e.g., maximal sprints) (Rommers et al., 2019), it is crucial to monitor PHV in order to explore sensitive periods of trainability that allow individualized training (Balyi and Hamilton, 2004).

Heart rate has been considered a useful predictor of aerobic performance changes over a soccer season (Krustrup et al., 2003; Impellizzeri et al., 2005). The HR_*max*_ (EaS–EnS period) highlighted improvements mainly from EaS to MiS and was related to VO_2m__*ax*_. In accordance with our results, significant effects (*p* < 0.05) on HR_*max*_ percentage in elite soccer players, after the EaS, were found by Kalapotharakos et al. (2011), whereas with reference to HR_*rest*_, it was reported to develop over the season with higher relation between EaS–EnS and MiS–EnS. The present study appears to confirm the close inverse relationship between physical fitness and HR reported in previous studies (Kingwell and Jennings, 1993; Rabbia et al., 2002). Previous research has also reported significant positive changes in HR after 4 weeks EaS, albeit in futsal players (Soares-Caldeira et al., 2014).

In the analysis of speed variables, a gradual improvement in LSS performance over the season was found and was highly related to the VO_2m__*ax*_ (EaS–MIS), as well as to the LSS (EaS–MiS and MiS–EnS). These results were also concordant with Dragijsky et al. (2017), who found significant improvements in running speed performance at the end of the season compared to the early season in young elite soccer players. The observed improvements in the present study may be attributed to the shuttle run and small-sided soccer games (anaerobic speed training with required change direction and LSS) implemented in the EaS. With regard to the accumulated load, a strong association between MALP (MiS–EnS) and HR_*max*_ (EaS–EnS) was found, indicating that the intermediate period of the season may be more positive for optimizing HR_*max*_. To the best of our knowledge, only one study (Clemente et al., 2020a) has analyzed the relationship between cardiorespiratory fitness and the accumulated training load in young soccer players. These authors found a large correlation between training load and improvements in aerobic power over time. The strong association obtained in the present study may be attributed to the natural morphological changes occurring during growth spurt, leading to the development of players’ motor skills. The results of the present study highlight the effects of training load and physiological changes over the season on performance, which should be considered by practitioners and researchers in their future work.

Predictive models are now ubiquitous in sports sciences research; nevertheless, most are developed to justify acute performance responses based on biomechanical and anthropometric measures (Barbosa et al., 2010; Morais et al., 2016) or even to predict aerobic capacity or maximal strength (Hoffman and Kang, 2003). Only one study, to the authors’ knowledge, provided indirect predictive models of the responses to different training program interventions in prepubertal children (Alves et al., 2020). Interestingly, the present study sought to understand and predict responses on fitness levels considering accumulated load, baseline levels, and PHV. First, analysis of VO_2m__*ax*_ showed significant results, where it was possible to predict the aerobic power levels of young soccer players during the season. Additionally, it was also found that both baseline VO_2m__*ax*_ and HR_*rest*_ were significant predictors of aerobic power levels. Contrarily, in the speed and physiological variables, respectively, there were no significant results. In fact, in LSS and LSM, poor predictors of speed were reported. It is conceivable that predictability would be more attainable if additional measurements were included, such as motor control, psychological aspects, or behavior genetics (Vandendriessche et al., 2011; Schutte et al., 2016). Nevertheless, despite the novelty of the present study, there are some limitations that should be addressed: (i) the number of included players was quite low, where adding more teams may have obtained more representative sample of the soccer young population; however, *a priori* power calculations indicated sufficient numbers were recruited, whereas *post hoc* power calculations indicated the achieved power; and (ii) the absence of psychological component evaluation, which could influence the motivation and performance.

## Conclusion

Aerobic and speed performance improved over the season and had a strong association with PHV. Moreover, the physiological variables demonstrated improvements from the EaS to EnS; however, improvements were more evident in the intermediate period, from EaS to MiS, and significantly associated with VO_2m__*ax*_. Thus, it is advisable that practitioners and researchers consider such changes in future work. Additionally, through multiple linear regression analysis, we were able to discern significant predictors of aerobic power levels of young soccer players based on their VO_2m__*ax*_ baseline and HR_*rest*_, whereas for speed and physiological variables, no significant results were found. Therefore, the results of the present study highlight the effects of training load and physiological changes over the season on performance, which should be considered by practitioners and researchers in their future work.

## Data Availability Statement

The raw data supporting the conclusions of this article will be made available by the authors, without undue reservation.

## Ethics Statement

The study was conducted in accordance with the latest version of the Declaration of Helsinki and approved by the Ethics Committee of the University of Isfahan with the ethical code number; IR.UI.REC.1397.181. Prior to study commencement, all players and their parents/guardians signed a written informed consent form. Written informed consent to participate in this study was provided by the participants’ legal guardian/next of kin.

## Author Contributions

HN, FC, and JP-G designed the study and drafted the manuscript. HN performed the experiments. HN, AA, CC, UG, and HZ participated in the data analysis and drafted the manuscript. HN, FC, UG, HZ, and JP-G revised the critical manuscript. All authors read and approved the final version of the manuscript.

## Conflict of Interest

The authors declare that the research was conducted in the absence of any commercial or financial relationships that could be construed as a potential conflict of interest.
